# The influence of water turbulence on surface deformations and the gas transfer rate across an air–water interface

**DOI:** 10.1007/s00348-024-03864-3

**Published:** 2024-08-28

**Authors:** Pim A. Bullee, Stefan Weichert, Astri Nore, Leon Li, Simen Å. Ellingsen, R. Jason Hearst

**Affiliations:** 1 Department of Energy and Process Engineering, Norwegian University of Science and Technology, Kolbjørn Hejes vei 2, 7034 Trondheim, Norway; 2 Department of Mechanical and Process Engineering, ETH Zurich, 8092 Zurich, Switzerland

## Abstract

We present experimental results of a study on oxygen transfer rates in a water channel facility with varying turbulence inflow conditions set by an active grid. We compare the change in gas transfer rate with different turbulence characteristics of the flow set by four different water channel and grid configurations. It was found that the change in gas transfer rate correlates best with the turbulence intensity in the vertical direction. The most turbulent cases increased the gas transfer rate by 30% compared to the low turbulence reference case. Between the two most turbulent cases studied here, the streamwise turbulence and largest length scales in the flow change, while the gas transfer rate is relatively unchanged. In contrast, for the two less turbulent cases where the magnitude of the fluctuations normal to the free surface are also smaller, the gas transfer rate is significantly reduced. Since the air–water interface plays an important role in the gas transfer process, special attention is given to the free-surface deformations. Despite taking measures to minimise it, the active grid also leaves a direct imprint on the free surface, and the majority of the waves on the surface originate from the grid itself. Surface deformations were, however, ruled out as a main driver for the increase in gas transfer because the increase in surface area is < 0.25%, which is two orders of magnitude smaller than the measured change in the gas transfer rate.

## Introduction

The transport of gaseous species across a gas–liquid interface is essential for numerous physics and engineering problems from carbon capture (Leung et al. 2014) to the ocean–atmosphere exchange of O$_2$ and CO$_2$; the former fosters life under the seas and the latter is largest carbon sequestration process on Earth (Sabine et al. 2004; Wanninkhof et al. 2009). At the root is a complex fluid mechanics problem that we are only starting to unravel (Jähne and Haußecker 1998; Yu 2019). A parameter that integrates the complex phenomena that govern this process into a single value is the gas transfer rate, *k*. Typically, *k* is defined as the ratio between the gas flux across the interface and the gas concentration in water, where the gas concentration is relative to its value at saturation, and has units distance per time. Focusing specifically on the air–water interface, previous laboratory studies have assessed how the gas transfer rate varies with turbulence in stationary systems by keeping the flow quiescent and generating turbulence within it (Herlina and Jirka 2008; Jirka et al. 2010; Variano and Cowen 2013) or flowing systems where the bulk Reynolds number is changed by varying the mean freestream velocities in the gas and/or liquid phases to influence the intensity of the bulk turbulent fluctuations (Turney and Banerjee 2013; Iwano et al. 2013). However, these two ideas have not been combined.

Parametrisations typically take on the form of a relation between the airspeed and the gas transfer, (Wanninkhof et al. 2009; Yu 2019). Nonetheless, these studies are limited in that one cannot control the conditions in the ocean, lakes, or atmosphere and thus the results represent the lumped effects of numerous mechanisms superimposed and interacting (Klaus and Vachon 2020). Field studies are without doubt necessary and insightful as they represent “what is actually happening”, but for an understanding of the individual underlying mechanisms, we turn to the controlled environment of the laboratory. This study thus presents an experimental approach to vary the turbulence intensity in a flowing system while holding the bulk Reynolds number constant, thereby decoupling the effects of the bulk flow processes and the turbulence intensity.

Danckwerts (1951) first proposed that in the presence of near-surface turbulence, a mechanism referred to as “surface-renewal” dominates the behaviour of *k*. Fresh, unsaturated parcels of fluid below the surface are constantly being transported to the surface by turbulence, where they can quickly absorb gaseous species from the air before being replaced by new fluid parcels. Furthermore, Henstock and Hanratty (1979) suggested that one also needs to consider multi-scaled turbulence farther down into the bulk flow, in addition to the turbulent transport near the surface, to accurately model the increase in *k* due to surface-renewal events. Assuming that there are bulk flows in both phases, there has been some disagreement as to whether it is the turbulence in the gaseous phase (e.g. McCready and Hanratty 1985; Komori et al. 1993) or the liquid phase (e.g. Coantic 1986; Jähne et al. 1987) that is more dominant.

Existing studies are enlightening. Herlina and Jirka (2008), Jirka et al. (2010), and Variano and Cowen (2013) produced turbulence in quiescent water boxes in order to measure the effect of turbulence on *k* and to facilitate an understanding of the dominant mechanisms. Herlina and Jirka (2008) produced water-side turbulence by oscillating a grid at the bottom of their tank; Jirka et al. (2010) put cooled air above their water to generate buoyant-convective mixing; and Variano and Cowen (2013) used a series of actuated jets to manipulate the turbulent conditions in their box.

In a numerical study, Herlina and Wissink (2014) found that *k* is dominated by the scales of the largest eddies in the bulk flow of their direct numerical simulation of a quiescent tank with isotropic turbulence. Furthermore, their results are in agreement with the surface divergence model of McCready et al. (1986) to a factor of 0.525. In general, these studies found that increasing the turbulence in the water increased *k* across the interface, but that the mechanism for this, and the ability of different models to predict the results, varied with turbulence intensity (or turbulent Reynolds number). These studies are insightful and give important information about the nature of mixing within the water, but are also limited in the achievable Reynolds number and the corresponding magnitude of the Reynolds stresses in the flow by two constraints of the set-up, namely that the interface is weakly deformed and that there is no bulk flow. These two constraints put limitations on the applicability of possible experiments with respect to field conditions (e.g. gas exchange processes in lakes, rivers, and oceans), where significant surface deformation and bulk flow can be present. It is imperative that studies reflecting field conditions are also performed.

**Fig. 1:**
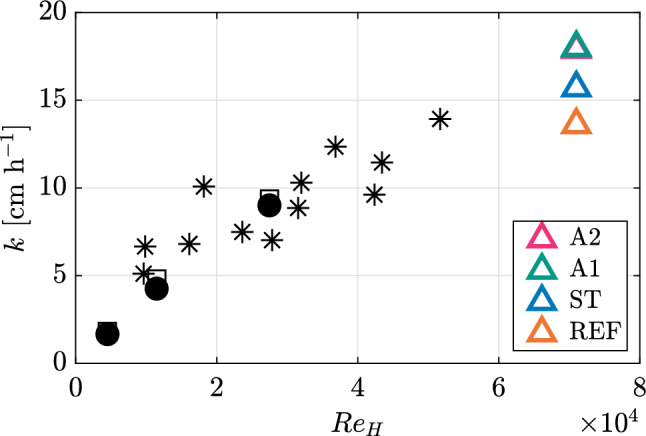
Example illustration of the relation between *k* and $Re_H = U_0H/\nu$. Data extracted from Eloubaidy et al. (1969) (asterix) and Turney and Banerjee (2013) (square and circle). For Turney and Banerjee (2013), squares represent the modified surface divergence model, while circles represent the measured values. The results from the present study are also shown, as triangles, to demonstrate the effects of freestream turbulence for the same $Re_H$

To overcome some of these limitations, experiments have also been performed in flowing systems. Such studies are numerous, but here we focus on a few illustrative examples. Turney and Banerjee (2013) designed a set-up where they could have flowing air over water, and also just flowing water. They measured the rate at which oxygen was transferred into the water via differential measurements at multiple locations. In general, they found that a modified version of the surface divergence model, a model that effectively considers both the intensity and the integral scale (representative of the separation between renewal events) of the flow, was able to predict *k* in their experiment after some empirical tuning. Figure 1 shows the resulting quasi-linear relation between *k* and the liquid phase bulk Reynolds number $Re_H = U_oH/\nu$, where *H* is the water depth, $U_0$ is the freestream velocity, and $\nu$ is the kinematic viscosity, for both their model predictions and measured values. Their results match well with the earlier findings of Eloubaidy et al. (1969), which are also shown in Fig. 1. Eloubaidy et al. (1969) showed that the quasi-linear relation extends into higher bulk Reynolds numbers. As a preview, our results are also included in Fig. 1 to foreshadow the effects of freestream turbulence for flows with the same $Re_H$. One should bear in mind that our experiment differs from the others in the figure in the respect that *U* is not the primary source of turbulence production in our case—the active grid is—so a similar behaviour of $k$ for varying $Re_H$ is not expected.

Turney and Banerjee (2013) also noted that “capillary waves are found to contribute to surface divergence but to have too short a time scale to cause interfacial gas transfer”. Xu et al. (2006) used an annular rig where they could create co- and counter-flowing systems of water and air. They found that gas transfer was dependent on the gradient of the vertical velocity fluctuations, but that the interaction was more complicated than simply parameterising the bulk characteristics. In an extreme case, Iwano et al. (2013) changed their velocity up to a 10-metre height equivalent velocity of 70 m/s and again found that *k* scales with both the mean velocity and corresponding fluctuations, but at these extreme velocities the surface is very deformed and the dominant mechanism shifts to being related to breaking and surface disintegration phenomena. Although the aforementioned studies span a wide range of speeds and experimental designs, they all increase the turbulent fluctuations by increasing the bulk velocity. Thus, they cannot decouple turbulence mechanisms from bulk mechanisms.

In order to achieve this, one must be able to generate the same bulk velocity while changing the turbulent parameters. This has been done in the past by changing bed roughness (Horoshenkov et al. 2013) or using bed-mounted ramps (Sanjou 2020) in open channel flows. However, these require significant physical changes to the set-up in order to create multiple data points. An alternative is to use an active grid (Makita 1991). These are devices where actuators are used to control an array of square wings at the inlet of a flow facility to create a transient blockage. By changing the pattern at the inlet, a certain degree of control over the produced turbulent parameters can be exerted (Larssen and Devenport 2011; Hearst and Lavoie 2015; Griffin et al. 2019; Neuhaus et al. 2020). This methodology is elegant in that a single set-up can be used to probe a variety of different turbulent flow conditions, replacing the need to change the entire bed or ramp geometry to change the turbulent parameters. Measurements in the turbulent flows produced by these devices show that the friction velocity along a rigid wall changes when the turbulence above the boundary layer is manipulated (Sharp et al. 2009; Hearst et al. 2018; Jooss et al. 2021), as well as the surface deformation (Savelsberg and van de Water 2008). Given these effects, it follows that *k* is likely to be influenced as well by the action of the active grid.

This study thus uses an active grid to manipulate the turbulence in the water of an open channel flow and study the impact on *k*. Particle image velocimetry (PIV) and laser Doppler velocimetry (LDV) are used to characterise the turbulent flow. Wave gauges and free-surface synthetic schlieren are used to characterise the surface deformations, and oxygen probes are used to measure the dissolved gas concentration evolution over time in the flow. This set-up provides the framework from which we investigate the impact of turbulence on gas transfer for fixed bulk flow properties.

Using this approach, we are able to investigate the fundamental mechanics of gas transport at an air–water interface regardless of the flow and turbulence properties in the air, as the turbulence levels within the water can be adjusted independently, unlike studies where wind is blowing over the water surface, generating turbulence and waves in the water. This fills an important knowledge gap, as gas transport models that are exclusively based on wind speed struggle to accurately predict the gas transfer velocity at low wind speeds ($U_{10} \lessapprox {3.7}{\mathrm{m \, s}}^{-1}$) (Crusius and Wanninkhof 2003) where the wind is no longer found to be the most important contributor to the near-surface turbulence that regulates the gas transfer velocity (Wanninkhof 1992). For example, wind over lakes typically falls within this low wind regime due to sheltering by the surrounding natural or urban elements, and wind speed-based prediction models were found to perform poorly in predicting gas transfer in lakes (Klaus and Vachon 2020). We therefore aim to contribute to a process level understanding of gas transfer achieved in a controlled laboratory environment.

## Materials and methods

### Water channel facility

**Fig. 2:**
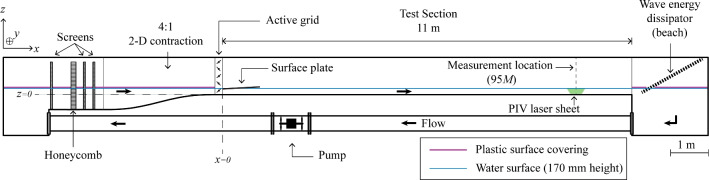
Side-view schematic of the water channel. Indicated are the flow direction of the water, as well as the pump and flow conditioning honeycomb and screens that drive and straighten the flow. The surface plate directly downstream of the active grid is in place to dampen the strongest water surface deformations caused by the active grid. Similarly, the beach is in place at the far end of the test section to dampen and reduce reflections of surface deformations. Outside of the test section, the water surface was covered by floating plastic sheets to ensure gas transfer only takes place in the test section where we control the flow on the water side

All experiments were conducted in the recirculating open water channel at the Norwegian University of Science and Technology (NTNU) (Jooss et al. 2021). A schematic of the facility is provided in Fig. 2. We only control the flow on the water-side of the facility. As it is an open channel, the stationary air above the water surface is in direct contact with the air in the laboratory. The streamwise (flow) direction is indicated by the direction of the positive *x*-axis, whereas the vertical direction aligns with the *z*-axis. Throughout, $(x,y,z)=(x_1,x_2,x_3)$ denote the streamwise, spanwise and vertical coordinates, with corresponding velocities $(u,v,w)=(u_1,u_2,u_3)$. The test section of the channel is made from glass for full optical access and measures 11.2  m  in length and 1.8  m  in width. For this study, it was filled with tap-water up to a height of $H = {170}\, {\textrm{mm}}\,$. The contraction in front of the test section has a 4:1 ratio, and an active grid is installed between the contraction and test section to control the turbulence of the flow.

**Fig. 3:**
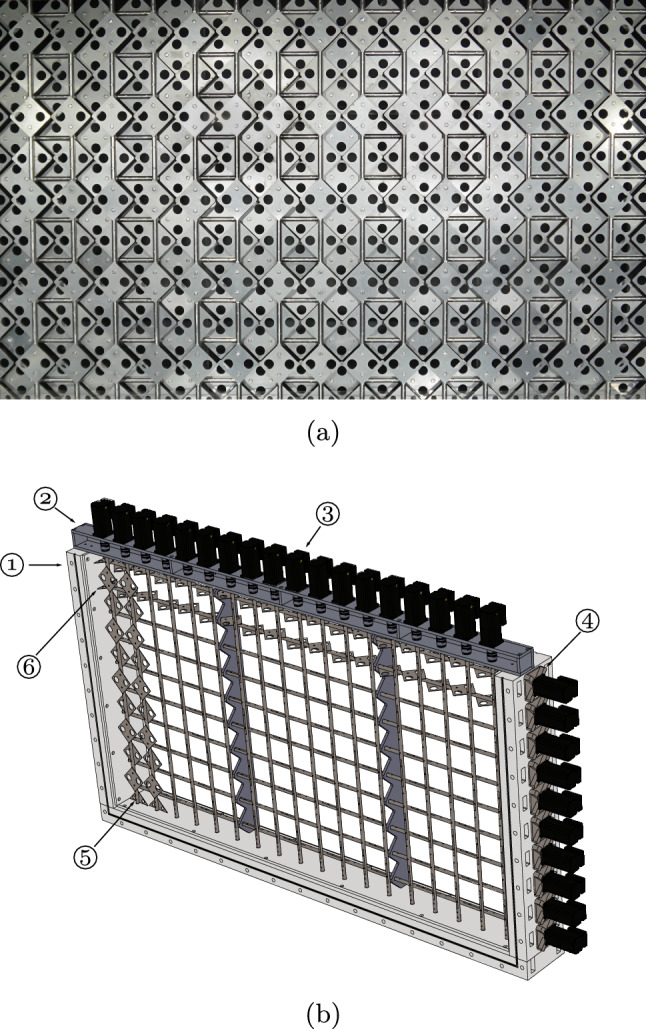
**a** Front view of the active grid with the space-filling wings fully closed. **b** 3D schematic of the active grid showing: (1) the acrylic outer frames, (2) the aluminium top frame, (3) the stepper motors, (4) the water-tight side motor mounts, (5) the vertical rods with wings, (6) the horizontal rods with wings. Note that only two of each of the horizontal and vertical rods are shown with wings, the other wings are hidden to avoid clutter in the schematic

The design of the active grid is based on the design of Makita (1991) and consists of 10 horizontal and 18 vertical bars, each with a diameter of 12  mm . The mesh length of the grid is defined as the centre-to-centre spacing of the bars, $M ={100}\, {\textrm{mm}}\,$. Figure 3 shows an image and a 3D schematic of the active grid. More details on both the facility and the active grid are presented by Jooss et al. (2021). As the water level in this study is 170  mm , only the bottom horizontal bar was used, while all vertical bars were used. The width of the active grid spans the entire cross section of the test section. Each active grid bar can rotate around its own length-axis and is connected to a stepper motor that controls its rotation. Attached to the bars are diamond-shaped wings with a diagonal of 100 mm that have holes in them to prevent them from fully blocking the flow. The wings spin with the bars, and by doing so, they interact with the flow that passes through the grid. As a result, turbulence is added to the flow, and the characteristics of the produced turbulence can be tailored by changing the actuation sequence of the wings; in this study, we use the same actuation approach as described by Hearst and Lavoie (2015). A 1-m-long acrylic plate with a thickness of 10  mm  was placed on the water surface directly downstream of the active grid, at the start of the test section, to dampen surface waves and air-bubble entrainment. The remaining 10 m of the water channel test section had a free surface. The water surface in the contraction and end section was covered with a floating plastic film to prevent air–water gas transfer in areas of the facility other than the test section.

### Test cases

Four different flow conditions were investigated in this study, set by a different status of the active grid. All cases had the same mean flow velocity $U_0 = {0.41}{\mathrm{m \, s}}^{-1}$ that was determined from laser Doppler velocimetry (LDV) measurements in the freestream region of the flow. We define the bulk Reynolds number as $\text {Re}_H=U_0 H/\nu$, where $H = {170}\, {\textrm{mm}}\,$ is the water height and $\nu$ is the kinematic viscosity. This Reynolds number has the same value $\text {Re}_H=71\times 10^{3}$ for all four cases. By using the direct forcing of the active grid we set the turbulence characteristics of the test cases. We define a turbulent Reynolds number as $\text {Re}_T = {u' L_{uu}}/{\nu }$ with $u'$ the streamwise velocity fluctuations and $L_{uu}$ the streamwise velocity integral length scale and give its value for each case in Table 1. The advantages of directly forcing the turbulence over indirect forcing methods are clear. Increasing the turbulence intensity by increasing the flow velocity will, for instance, result in larger bulk Reynolds numbers, and shorter transfer times for the liquid travelling through the test section. Using passive grids necessitates changing the experimental set-up for each test case. Therefore, using the active grid to control the turbulence in the flow in a single set-up allows for a direct comparison of the different test cases. Here, we employ two active grid cases (A1 and A2), a static grid case (ST), and a no-grid case (REF). The no-grid case is the reference flow in the absence of a grid installed in the water channel. For the static grid case the grid was installed in the channel, but kept in a fully open position with its wings aligned with the flow. For the two active grid cases, the actuation modes of the motors that control the rotation of the active grid bars follow a fully random protocol (Hearst and Lavoie 2015). This means that the rotational velocity, period and acceleration were varied randomly within a predefined range of values, set by a top-hat distribution. The rotational velocities $\Omega \pm \frac{\Omega }{2}$ for the two active grid cases were ${0.50 \pm 0.25}{\textrm{Hz}}$ for A1and ${0.05 \pm 0.025}{\textrm{Hz}}$ for A2. This resulted in a range of flow characteristics for the four different test cases given in Table 1.

**Table 1:** Overview of the different test cases. $\Omega$ gives the mean rotational frequency of the top-hat distributions with outer bounds $\Omega \pm \frac{\Omega }{2}$ that define the rotational velocity of the grid bars. The turbulence intensities for the streamwise and vertical components $u'/U$ and $w'/U$ are from the PIV measurements. The largest length scales in the flow are represented by the value for $L_{u_iu_i} / H$ calculated from the PIV data at a height of 0.75*H*. The water temperature and the standard deviation of the surface elevation are given by *T* and $\eta ',$ respectively. The gas transfer coefficient is given by *k*. For all cases, the mean streamwise velocity $U_0 = {0.41}{\mathrm{m \, s}}^{-1}$, giving a bulk Reynolds number $\text {Re}_H=71\times 10^{3}$

Case	Grid	$\Omega$ [Hz]	$u'/U$ (%)	$w'/U$ (%)	L$_{uu} / H$	L$_{ww} / H$	$\text {Re}_T$	*T* [$^{\circ }$C]	$\eta '$ [mm]	*k* [${\mathrm{cm \, h}}^{-1}$]
REF	None	–	2.7	1.9	0.42	0.05	760	19.2	0.1	13.6
ST	Static	0.00	2.8	2.1	0.32	0.03	600	19.6	0.5	15.7
A1	Active	0.50	4.6	3.0	0.55	0.07	1750	19.5	2.3	18.0
A2	Active	0.05	7.1	3.1	1.52	0.07	7300	19.2	1.8	17.9

### Oxygen probe measurements

At the start of each measurement, the water in the facility was depleted of dissolved oxygen by adding sodium sulphite which reacts with the dissolved oxygen to form sodium sulphate. In this way, the dissolved oxygen concentration is dropped to zero. Once all the sodium sulphite has reacted with oxygen, the level of dissolved oxygen will again start to increase until it is restored at its saturation value in the water. This allows us to observe the full evolution of the gas transport of dissolved oxygen into water from a concentration of zero to a fully saturated concentration. This approach has been used previously in other large recirculating water facilities, e.g. Sanjou et al. (2017); Sanjou (2020). For the different test cases introduced in Sect. 2.2, we record the evolution of oxygen concentration with time at $x = 95M$ downstream of the active grid, for up to 60 h after reaching zero dissolved oxygen concentration. To this end, we sampled the dissolved oxygen concentration at a frequency of 1Hz using a 0.8-mm-diameter extra-fine PreSens Microx 4 optical dipping probe with a measurement range of 0-100% dissolved oxygen, and average over 15 min intervals to obtain a time series of the reabsorption of oxygen by the water. Due to its small size, the probe had a minimal effect on the flow. The calibration was performed using a conventional two-point calibration. The currently reported oxygen concentration data are acquired sufficiently far from the concentration boundary layer that it can be considered the bulk, as was reported by Nore (2022). The temperature of the water in the experimental facility and the atmospheric pressure in the laboratory were recorded in parallel with the oxygen concentration, and used to calculate the saturation concentration of dissolved oxygen for the normalisation of the measurements.

### Flow characterisation

**Fig. 4:**
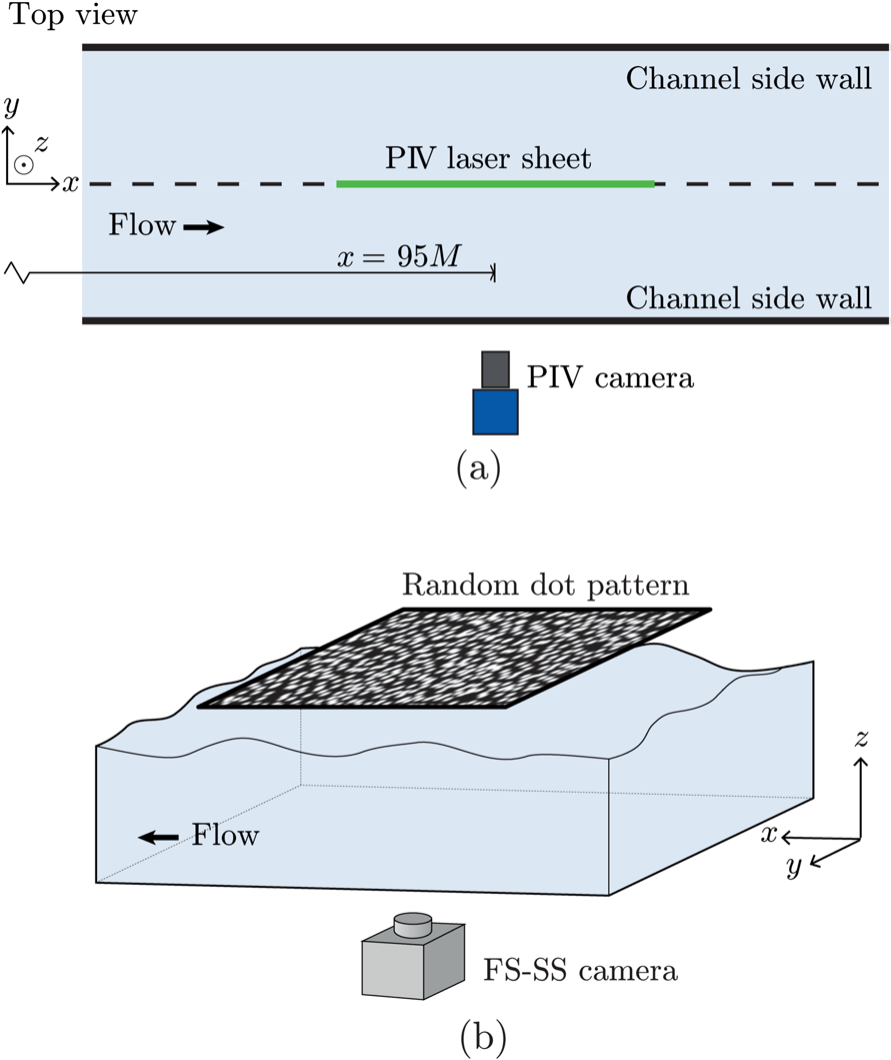
**a** Top-view schematic of the PIV set-up. The laser is placed under the water channel, and illuminates the flow through the glass bottom wall. **b** The free-surface synthetic schlieren set-up

We used a combination of PIV and LDV to characterise the flow. The flow was seeded with 40 $\mu$m Dynoseeds TS40 6317 spherical polystyrene particles with a density of 1050${\textrm{kg m}}^{-3}$. For the PIV measurements, a Litron Nano L200-15 Nd-YAG dual-pulse laser was used to generate a $\sim$ 1-mm-thick light sheet that illuminated the particles. The laser sheet was oriented in the streamwise direction at the centre of the channel 95*M* downstream of the active grid, the same position as where the oxygen probe was installed, see also Fig. 4a. A 25M LaVision Imager MX 25 M camera paired with a 100  mm  ZEISS Milvus 100 M macro lens was placed alongside the water channel and used to record the particle images. The field of view was ${170}\, {\textrm{mm}}\, \times {170}\, {\textrm{mm}}\,$ in the streamwise-wall-normal plane, which allows us to resolve the flow over the full water height. For each test case, 2000 independent image pairs were acquired at a sampling rate of 2Hz with an inter-frame time of 1125$\mu$s, which is sufficient to statistically converge the first- and second-order velocity statistics.

LaVision DaVis 10 was used to collect and process the data. In pre-processing, the minimum value of each pixel in the time ensemble was subtracted from the images to decrease background noise. A $5 \times 5$ Gaussian profile was used to subtract a sliding average. Processing was done with a decreasing interrogation window size with a $48\times 48$ window size and ${50}{\%}$ overlap for the final pass. The vector spacing in the resulting velocity fields is approximately 0.8 mm in both the streamwise and wall-normal (vertical) directions.

LDV measurements were also performed 95*M* downstream of the active grid in the centre of the channel, 30 mm below the free surface. A continuous laser with a wavelength of 514.5 $\mu$m was used in combination with a 60-mm-FiberFlow probe from Dantec Dynamics to measure streamwise velocities. The mean sampling rate varied between the test cases in the range ${80}{\textrm{Hz}} \le f_s \le {270}{\textrm{Hz}}$, and a minimum of 100 000 samples were taken for each test case. To estimate the power spectral density of the measured velocities, an arrival-time quantisation method was used, following the work of Damaschke et al. (2018).

### Characterisation of free-surface motion

Surface height variations were monitored using a resistive wave probe from HR Wallingford. It was mounted directly next to the dissolved oxygen probe $x= 95M$ downstream of the active grid. The surface height was measured during the full course of each experiment with a recording rate of 100Hz.

For better characterisation of the free-surface motion, we employed an adaptation to the free-surface synthetic schlieren (FS-SS) method of Moisy et al. (2009), which builds on the schlieren effect through the reconstruction of a refracted image viewed through a disturbed interface. We used a Photron Fastcam Mini WX100, paired with a Sigma 105 mm macro lens, placed under the water channel 95*M* downstream of the active grid in the centre of the channel. The camera was facing up, and imaged a pattern of randomly organised dots mounted above the free surface, viewing through the glass bottom window, the water and the air–water interface. The set-up is sketched in Fig. 4b, and a more detailed description can be found in Weichert (2024). Refraction of light where the surface is not flat causes the dot pattern that the camera records to be distorted. Using an undisturbed image of the dot pattern imaged through a flat air–water interface, the disturbed interface can be reconstructed (Moisy et al. 2009). For each test case, three independent image sequences of 1500 images were recorded, each at a frequency of 50Hz, such that the surface motions were temporally resolved. The recorded images were cross-correlated with the undisturbed reference image using the LaVision Davis 10 software package, with $32\times 32$ window size and a ${50}{\%}$ overlap for the final pass. The resulting gradient fields were analysed with an in-house developed code to give the surface elevations. The sampled surface area has dimensions of ${165}\, {\textrm{mm}}\,\times {165}\, {\textrm{mm}}\,$ divided over $128 \times 128$ pixels. This gives a spatial resolution of 1.29  mm  in both the streamwise and spanwise directions.

## Results and discussion

### Dissolved oxygen concentrations

**Fig. 5:**
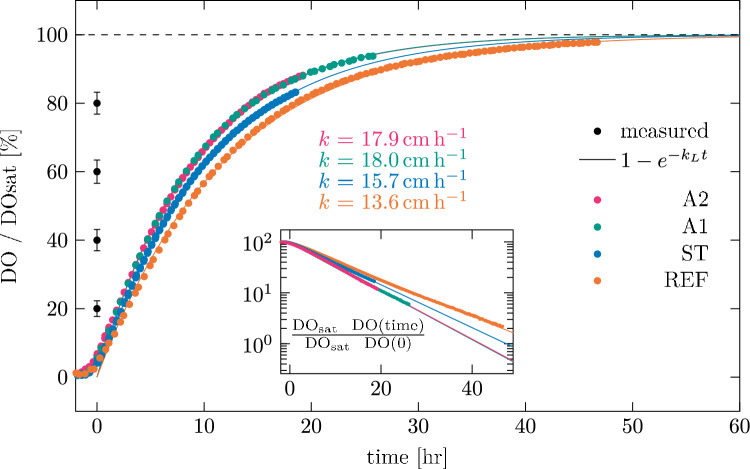
Evolution of the bulk oxygen concentration in time for the different test cases. Only every third point is plotted for clarity, and each point is an average of a 15 min interval. Typical uncertainties in the measurement data are indicated using the error bars for different values of $\text {DO}/\text {DO}_\text {sat}$. To highlight the exponential characteristic of the measurement data, a logarithmic scale is used for the inset plot

Figure 5 shows the dissolved oxygen concentrations $\text {DO}$ versus time for the different test cases. The $\text {DO}$ was measured in grams per litre, and shown normalised with its value at saturation $\text {DO}_\text {sat}$ computed from a look-up table based on the works of Benson and Krause Jr. (1980, 1984) The starting time, $t = {0}$, is set using a threshold value for the difference in dissolved oxygen level between two consecutive measurements. The markers indicate the mean value that was measured over a period of 15  min. The measurement uncertainty is determined based on the uncertainty in the measured values as specified by the manufacturer, the standard deviation associated with the plotted mean value and the repeatability based on a comparison of four realisations of the most turbulent case A2. For readability, we indicate the measurement uncertainty for four different dissolved oxygen concentrations on the left side of the figure using error bars. We identify a characteristic region in which the oxygen concentration scales as $1-e^{-k_L t}$, where $k_L$ is the gas transfer rate coefficient specific for the experiment. We fit this to our data for $k_L$ over the time interval between 20% and 80% oxygen saturation and find these fits to align well with the measurement data. The fits to the data are included in Fig. 5, with the values of the generic gas transfer coefficient *k* also given in the same figure. From the weighted least square fitting using the determined measurement uncertainty, we find the uncertainty in *k* to be about 1%.

This generic value for the gas transfer rate coefficient $k=k_L \times H_\text {eq}$ is independent of the surface area-to-volume ratio, as $H_\text {eq} = {162}{\textrm{cm}}$ is the equivalent water height defined as the ratio between the water volume and the area of the free surface exposed to the atmosphere across which the gas transfer takes place. The computed values of *k* are reported in Table 1. We deliberately neglect the effect that surface deformations have on the area of the free surface, as we will demonstrate later in Sect. 3.3 that the area increase is negligible. We will further investigate the effect that the state of the grid in the different test cases had on the flow in Sect. 3.2 and will also comment on how this affects the free-surface topology in Sect. 3.3.

### Turbulence statistics and flow measurements

**Fig. 6:**
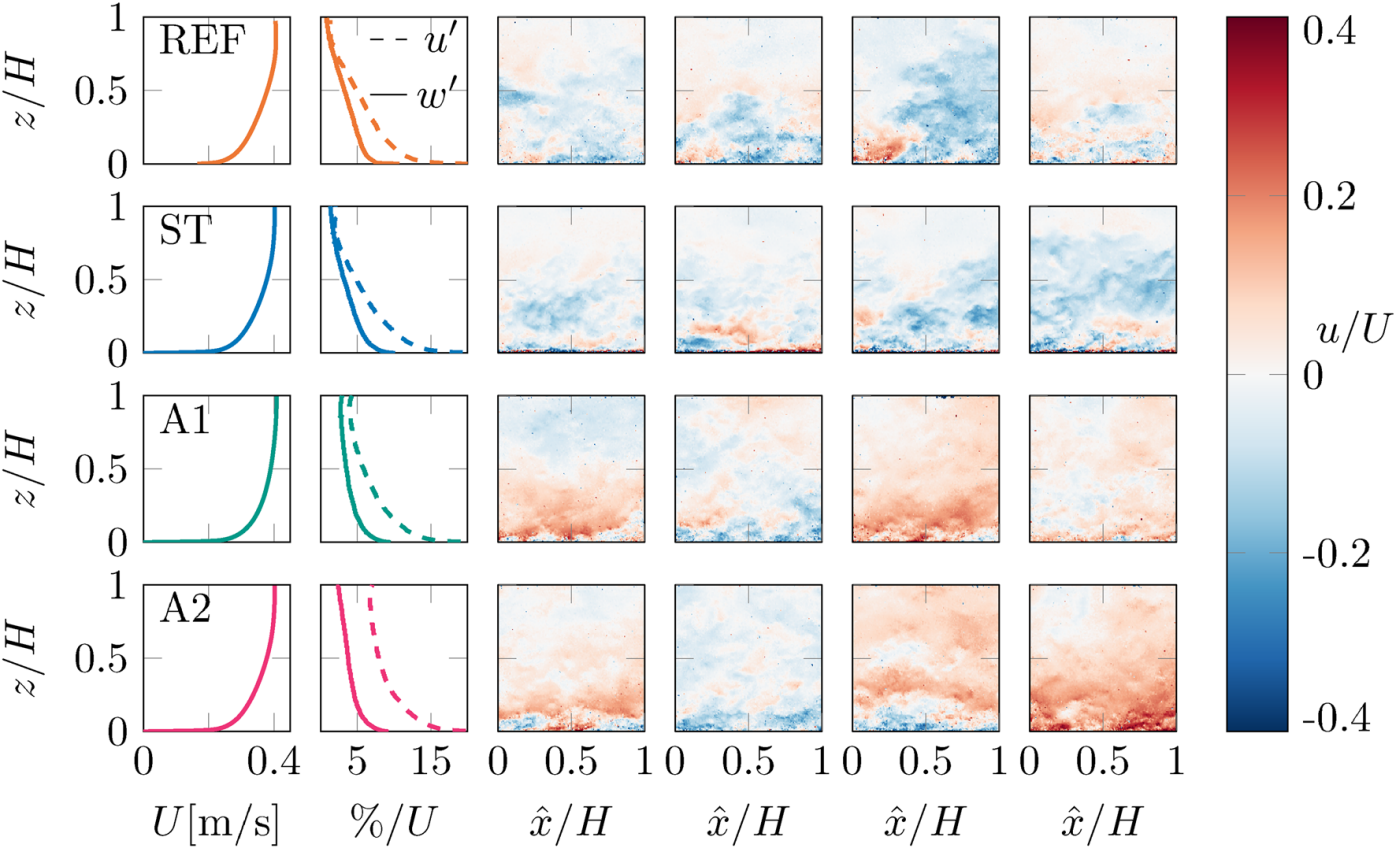
Plots of instantaneous streamwise velocity fluctuations $u(x,z,t)$ normalised with the mean velocity $U(x,z)$ from PIV measurements. Four examples are shown for each case chosen at random, and profiles of the mean streamwise velocity $U$, and the turbulence intensities $u'/U$ and $w'/U$ calculated using the full dataset are provided on the left. The effect of the active grid is most clearly seen where $z>0.5H$ with the appearance of turbulent structures and increasing $u'/U$ and $w'/U$. The data were acquired at $x = 95M$ in the centre of the channel; the same location as the oxygen concentration measurements. The ${\hat{x}}$-coordinate indicates the streamwise position inside the field of view of the camera

We present instantaneous snapshots of the fluctuating streamwise velocity fields. The turbulence intensity fields are defined as the standard deviations of the streamwise and vertical velocity fields $\mathcal {U}(x,z,t)$ and $\mathcal {V}(x,z,t)$ relative to the mean streamwise velocity field $U(x, { z } )$. From both, we calculate their streamwise averaged profiles $u'/U$ and $w'/U$, and shown them in Fig. 6 together with the mean streamwise velocity profile. From the profiles of $u'/U$ and $w'/U,$ it is clear that the turbulence intensity changes between the different grid cases, as quantified in Table 1 by the values averaged over the top half of the channel. The effect of the active grid is also most noticeable in the top-half of the flow, as is observed in the instantaneous snapshots of the flow from the appearing turbulent structures.

The turbulence intensities in both streamwise and vertical direction are only slightly increased for the static grid case ST compared to the no-grid case REF at $x=95M$. This is characteristic for static grid turbulence in water channel facilities that naturally have relatively large levels of background turbulence. Moreover, at 95*M* downstream of the grid, one would expect most of the introduced turbulence to have decayed into the background for the static case. Furthermore, this far downstream the majority of the flow is populated by the boundary layer, see Fig. 6, and also (Jooss et al. 2021) for boundary layer measurements in the same facility. When comparing the two active grid cases, A1 and A2, we find a significant difference in streamwise turbulence intensity, but very similar vertical turbulence intensities, as is reflected by their values in Table 1 and also visible in the profiles in Fig. 6.

In relation to gas transfer, which in essence can be thought of as a one-dimensional mechanism of transport from the free surface to the deeper layers in the liquid, this tells us that the turbulence in the vertical direction may be a more relevant parameter than the streamwise turbulence. We find small increases in both $u'/U$, $w'/U$, and *k* from REF to ST, significant increases in all three quantities from ST to A1, and when going from A1 to A2 we find a big change in $u'/U$ but essentially no change in $w'/U$, and correspondingly no significant change to the gas transfer rate.

**Fig. 7:**
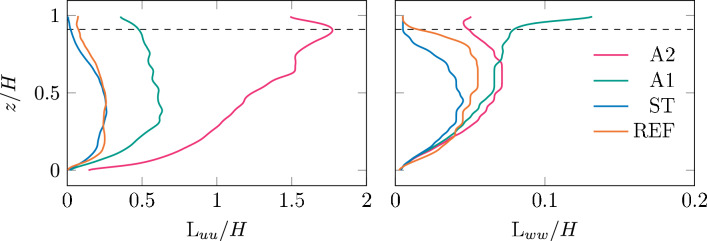
Streamwise integral length scales $L_{uu}$ and $L_{ww}$ for the streamwise and vertical velocities from PIV. The integral length scale is defined as the distance over which the velocity autocorrelation $R_{u_i u_i}$ reduces to 0.5. For case A2, $R_{u u}$ was extrapolated using exponential fitting since it did not reduce to 0.5 within the width of the field of view of the PIV. The dashed line indicates a depth of 1.5cm below the mean water height *H*. As the water level is fluctuating in time due to a varying blockage of the active grid and from waves passing by, nothing conclusive can be said about this region.

To gain understanding of the influence of the grid on the characteristic largest length scales of the flow, we present in Fig. 7 integral length scales versus height for the different cases. The integral length scales $L_{uu}$ and $L_{ww}$ were defined as the streamwise distance over which the velocity autocorrelation defined as

1 $$\begin{aligned} R_{u_iu_j}(z) = \left. \frac{\left\langle u_i(x,z)u_j(x+\Delta x,z\right\rangle }{\left\langle u_i(x,z)u_j(x,z)\right\rangle } \right. \end{aligned}$$

from PIV reduces to a value of $R_{u_iu_j} = 0.5$. The values for $L_{u_iu_i}$ at a height of $z = 0.75 H$ are provided in Table 1 to be representative of the region in the flow that is most influenced by the active grid. This is particularly strong in the top part of the channel, see, for instance, also (Jooss et al. 2021).

Similar to what we found for the turbulence intensities, the values for $L_{u_iu_i}$ are very similar for no grid and static grid, indicated REF and ST. Both $L_{uu}$ and $L_{ww}$ increase when activating the grid and grow to double their length or more for case A2. Comparing the length scales to the gas transfer coefficients, we find the value of *k* increases with both $L_{uu}$ and $L_{ww}$ in Table 1 when comparing REF and ST to A1 and A2, but between A1 and A2 the trend is opposite for $L_{uu}$, and likewise for REF and ST with $L_{ww}$. Hence, no overall trend between $L_{u_iu_i}$ and *k* is found.

The turbulence statistics presented here are in line with expectations, as for active grid-generated turbulence it is well known that for slower rotation rates the streamwise turbulence intensity and the characteristic largest length scales of the flow increase (Hearst and Lavoie 2015). Moving farther upstream, however, it is expected that the turbulence intensities will increase for the same active grid actuation sequence, whereas the largest length scales found in the flow will be shorter. An example of this in the same facility was presented by Jooss et al. (2021).

**Fig. 8:**
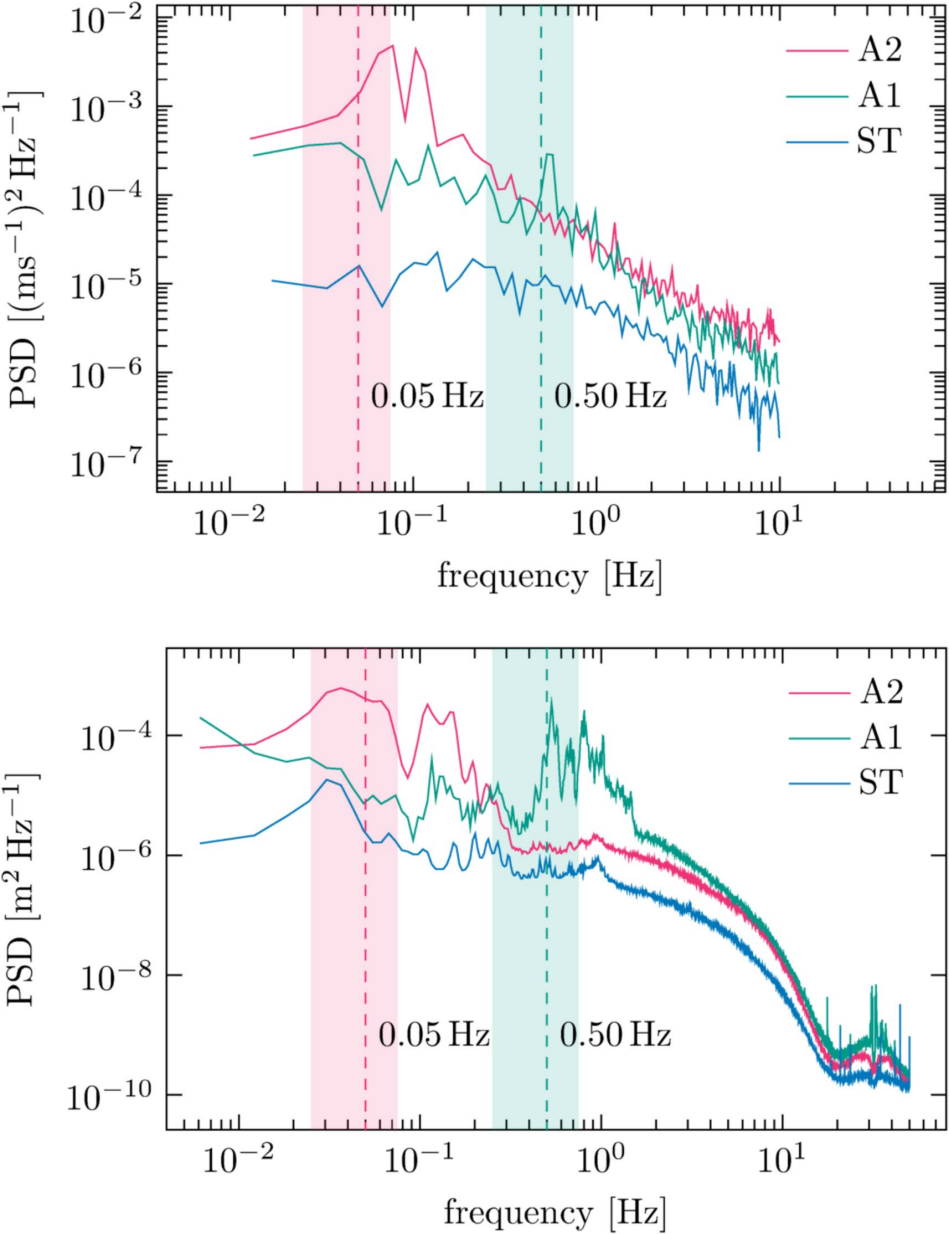
Top: Power spectral density (PSD) for the streamwise velocities from LDV measurements at $x = 95M$. The coloured bands identify the range of actuation frequencies of the active grid for cases A1 and A2, with the dashed line at the mean of the range. Bottom: PSD of the surface deformation $\eta (95M,0,t)$ from the wave probe at $x = 95M$

The actuation sequence of the active grid leaves a clear imprint on the flow. This is illustrated in Fig. 8, where we plot the power spectral density of the streamwise velocities as measured by LDV. The forcing frequencies of the two active grid sequences are indicated in the plot and are in the vicinity of the peaks in the corresponding spectra from both the velocity and the wave probe data. This gives rise to the question if these peaks in the wave spectra are related to the turbulence in the flow or to the action of the active grid, upon which we will expand in the following sections.

### Wave statistics and surface measurements

We discuss the 2+1-dimensional measurements of the free surface from synthetic schlieren data. First, we provide some notes on how to interpret wavenumber-frequency spectra of free-surface motion, before analysing our measured spectra.

#### On the interpretation of free-surface elevation spectra

The action of the active grid results in two physically distinct surface features which our measurements pick up: surface imprints of large turbulent structures convected with the mean flow and propagating waves. The relative presence of the two classes of features provides a significant amount of information about the type of fluid flow is present close beneath the surface. Gentle surface waves are associated with approximately irrotational flow wherein fluid parcels move in orbital paths, a type of motion which does not renew the surface. Imprints of turbulent eddies, on the other hand—frequently seen in riverine flow—are intimately connected to the upwelling of unsaturated water from the bulk (Muraro et al. 2021; Babiker et al. 2023), to the extent that they can potentially be used for remote sensing of gas flux in such environments (Dolcetti et al. 2022).

**Fig. 9:**
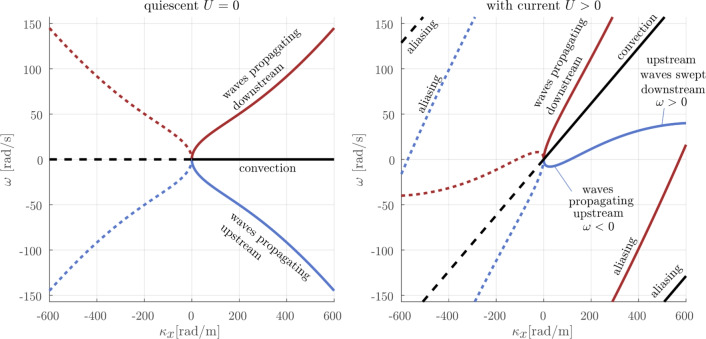
Schematic of where spectral intensity $|{\tilde{\eta }}(\kappa _x,0,\omega )|$ is to be expected from the synthetic schlieren data in the streamwise-temporal $(\kappa _x,\omega )$ plane, i.e. $\kappa _y=0, \kappa = |\kappa _x|$. Solid lines may be interpreted as the physical features and dashed and dotted lines as features, redundant due to point symmetry about the origin. Freely propagating waves produce intensity near the dispersion relation (2), while signatures of turbulent eddies convected approximately with the mean flow velocity lie near the diagonal line $\omega =\kappa _xU$. For waves, the phase velocity in the *x*-direction is $\omega /\kappa _x$. In the corners, periodic (in $\omega$) continuations of the curves appear due to aliasing, i.e. frequencies higher than half the sampling frequency cannot be resolved and appear again, offset by the sampling frequency (50 Hz, $\omega _\textrm{max}\approx 157$ rad/s)

Transforming the surface motion $\eta (x,y,t)$ into a Fourier spectrum ${\tilde{\eta }}(\kappa _x,\kappa _y,\omega )$, the two types of surface motion appear in different ways. We consider here a spectrum in the streamwise-temporal $(\kappa _x,\omega )$ Fourier plane, $\kappa _y=0$ which contains the most useful information. A principle sketch of the spectral manifestations of surface waves and convected features is shown in Fig. 9a and similar analysis is briefly described by Kidanemariam and 2020 .

The redundancy inherent in a complex representation of a real function gives a spectrum which is point-symmetrical about the origin. It is most instructive for our purposes to choose to consider the half-plane, $\kappa _x>0$, to represent the “physical” surface fluctuations (spectral intensity near the solid curves) and the left half, $\kappa _x<0$, the redundant mirror image (dotted lines). Negative values of $\omega$ means the phase velocity $\omega /\kappa _x$ in the laboratory frame of reference is in the negative *x*-direction, i.e. upstream in the laboratory system. Likewise, spurious spectral intensity appears at high temporal and spatial frequencies due to aliasing (dashed and dotted lines in upper left and lower right corners).

Freely propagating surface waves on a steady current, *U*, in the positive *x*-direction must satisfy the well-known dispersion relation

2 $$\begin{aligned} \omega ({\varvec{\kappa }}) = \pm \sqrt{g\kappa + \sigma \kappa ^3/\rho } + U \kappa _x, \end{aligned}$$

i.e. the relation between the spatial frequencies $\kappa _x$, $\kappa _y$ and the temporal frequency $\omega$, where $\sigma /\rho$ is the kinematic surface tension coefficient, ${\varvec{\kappa }}=(\kappa _x,\kappa _y)$ and $\kappa =|{\varvec{\kappa }}|.$ (Indeed, extracting the dispersion curve from the peak in the surface wave spectra is a common way to measure ocean currents remotely, e.g. Stewart and Joy (1974); Smeltzer et al. (2019).) In consequence, their signal in a wavenumber/frequency plane will lie close to the curves $\omega ({\varvec{\kappa }})$ satisfying this relation. Note that while the negative-signed branch of the dispersion relation (2) in the $(\kappa _x>0,\omega )$ plane are waves propagating in the upstream direction in a frame of reference moving with the mean flow, it becomes positive when *U* is sufficiently large and hence represents waves whose phase still moves in the downstream direction. These waves, whose phase tries to propagate upstream, are not fast enough to do so and are swept downstream.

Spectral manifestations of surface features convected exactly with the mean flow *U*, however, will lie on the straight line $\omega (\varvec{\kappa }) = U \kappa _x$, shown as a straight black line in Fig. 9. In reality, the spectral imprints are broadened around these curves for a number of reasons: measurement uncertainty, spectral leakage and the physical facts that waves are locally impeded or assisted by turbulent flow fluctuations, and that long-lived turbulent features are not convected at exactly the mean flow velocity. Fast turbulent fluctuations will appear as a broadly distributed “noise” signal across the spectrum. Moreover, wave *energy* only propagates upstream if the group velocity $\textrm{d}\omega /\textrm{d}\kappa _x$ is negative, true only for very small values $\kappa _x\lesssim \frac{1}{4} gU^{-2}\approx 15$ rad m$^{-1}$, smaller than we can spatially resolve with our field of view, hence we can say nothing about the relative wave content in the corresponding free-surface motion frequency range $-12$ rad s$^{-1}\lesssim \omega <0$ from our synthetic schlieren data. We cannot preclude that some surface signal represents long (wavelength $\gtrsim 44$ cm) upstream propagating waves, e.g. due to reflections from the end of the channel. All signal in our 3D spectra which can be clearly identified as (mostly) due to waves thus represents waves whose energy travels downstream and hence cannot have been generated by a source farther downstream than where the measurements were taken.

#### Free-surface motion results

**Fig. 10:**
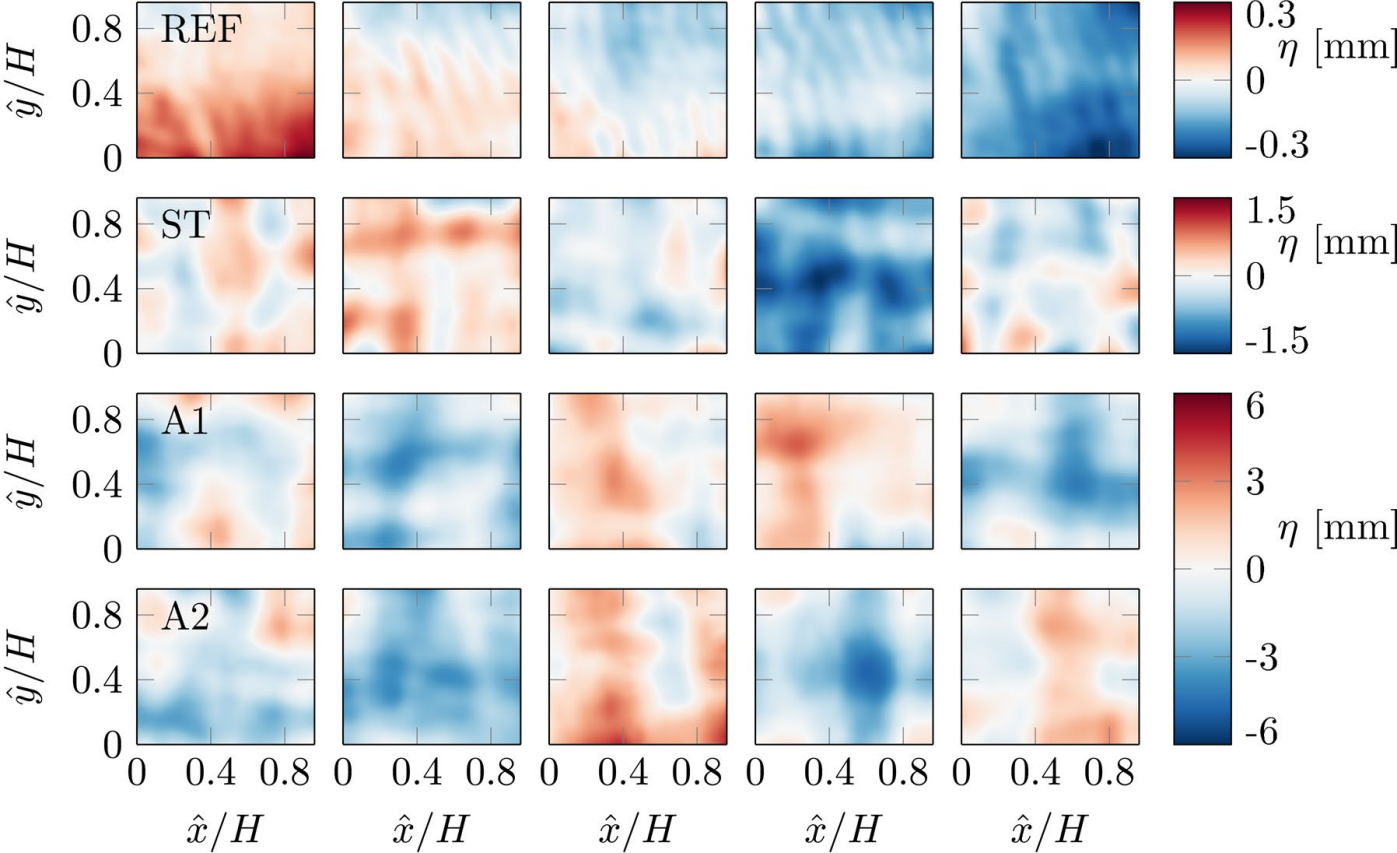
Instantaneous surface height fluctuations from synthetic schlieren measurements. Five examples chosen at random are shown for each case. The data were acquired at $x = 95M$ in the centre of the channel; the same location as the oxygen concentration measurements. The ${\hat{x}}$ and ${\hat{y}}$-coordinate indicates the streamwise and spanwise positions inside the field of view of the camera. Note the change in colour scale between different cases

A qualitative impression can be gained from instantaneous images of the free-surface motion shown in Fig. 10, acquired from synthetic schlieren measurements. It is clear that when the grid is used to produce turbulence, the surface waves also change. For REF, in the absence of the grid, we conclude from visual inspection that the surface disturbances are dominated by wrinkles originating from the joints that connect the glass windows of the channel wall. For the (active) grid cases, the surface is clearly composed of a larger variety of surface deformations of different wavelengths and larger amplitudes. The standard deviation of the surface height $\eta '$ differs between cases and is far higher for all cases where the grid is active, as listed in Table 1.

With the increasing deformations of the surface, the total free-surface area also increases, a possible reason for the increased gas transfer rate, which is proportional to surface area. A straightforward analysis of the change in free-surface area for the different cases shows, however, that the increase in free-surface area is negligible compared to the increase in gas transfer rate. For the most wavy case A1, the relative increase in the free-surface area compared to a flat surface,

3 $$\begin{aligned} \Delta \mathcal {S} = \frac{1}{\mathcal {A}}\left\langle \int _\mathcal {A}\bigl (\sqrt{1+|\nabla \eta |^2}-1\bigr )\textrm{d} x\textrm{d} y\right\rangle , \end{aligned}$$

(with $\mathcal {A}$ the measurement area in the *xy*-plane) is less than 0.25%. Thus, the increase in free-surface area is ruled out as a main cause for the change in *k* reported in Table 1.

**Fig. 11:**
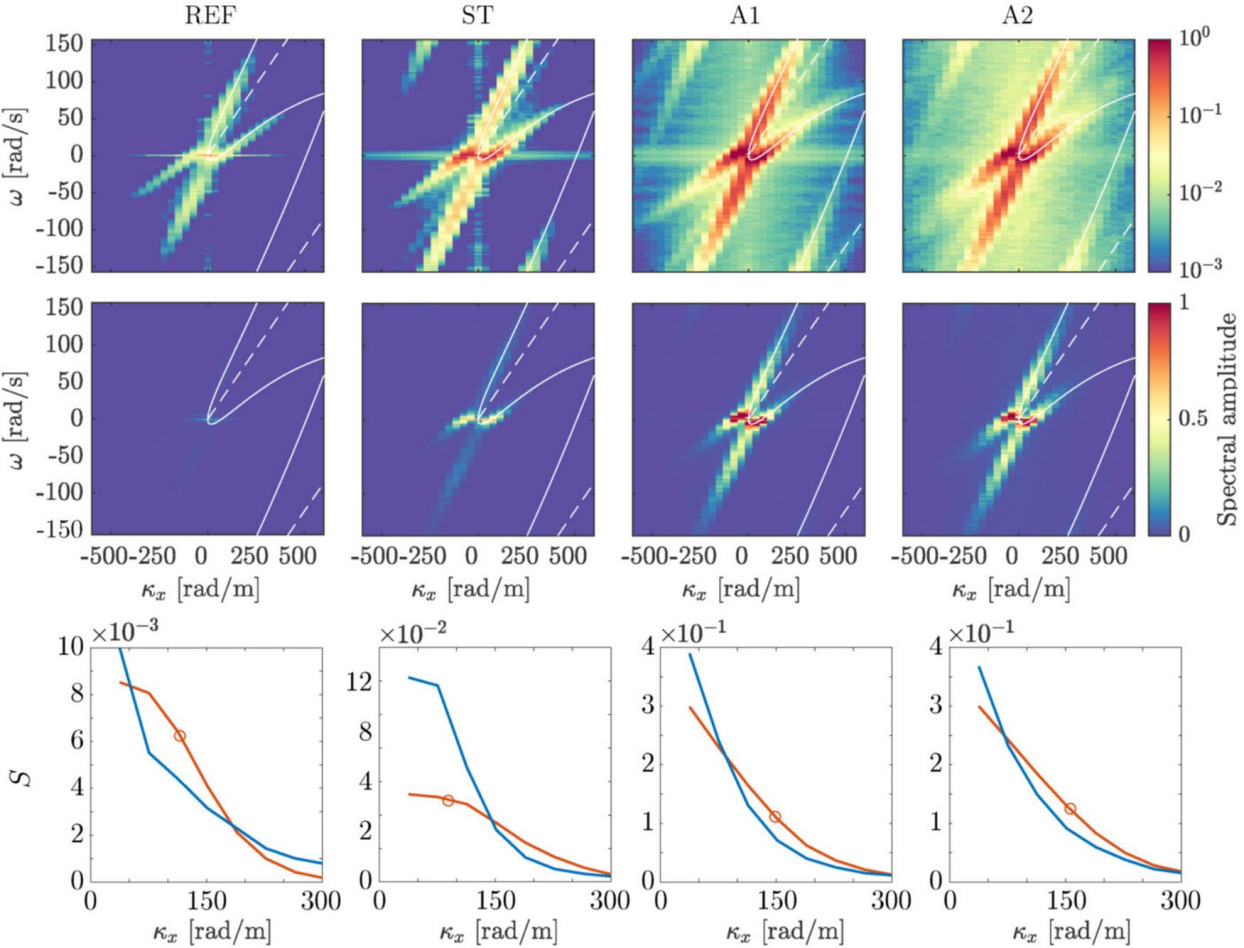
Spectra of synthetic schlieren data $\nabla \eta$, for the four test cases. Top row: log-scale $\kappa _y=0$ slice of 3D spectra, wave components travelling parallel to the channel. White lines indicate dispersion and convection curves as shown in Fig. 9. Middle row: linear scale of top row. Bottom row: spectral intensity along the upper (red line, marked with a circle) and lower (blue line) surface wave dispersion branches, corresponding to “$+$” and “−” sign in equation (2), respectively; colours match those in Fig. 9)

In Fig. 11, we plot the gradient spectrum $|\textrm{FFT}\{\nabla \eta (x,0,t)\}|$ where “FFT” means the numerical fast-Fourier transform in both time and space, $(x,t)\rightarrow (\kappa _x,\omega )$. We use $\nabla \eta$ rather than $\eta$ since the gradient is what we directly measure and hence is less noisy; the qualitative aspects, however, are similar as, formally, $\textrm{FFT}\{\nabla \eta (x,y,t)\}=\textrm{FFT}\{\eta (x,y,t)\}{\varvec{\kappa }}$. Spectra are shown in logarithmic (top row) and linear (middle row) scale, respectively. It is immediately clear that the dominating contribution comes from freely propagating surface waves. An enhanced spectral signal along the convection line associated with structures convected with the mean flow is discernible only in logarithmic scale, being more than an order of magnitude smaller.

Figure 8 shows the power spectral density (PSD) of both the streamwise velocity (top panel) and the time-varying free-surface elevation (bottom panel) at a point 95*M* downstream of the grid. We make several observations. First, the spectral peaks of the turbulent velocity at the grid’s actuation frequencies in cases A1 and A2 display corresponding peaks in the surface motion. There is significant wave energy also in the frequency half-decade above forcing, perhaps because of wave turbulence (a consequence of nonlinear wave–wave interactions, not to be confused with wave turbulence interaction—waves interacting with sub-surface turbulence); see, for example, Falcon and Mordant (2022). Case ST (static grid) shows less peaked behaviour as well as much lower surface fluctuations overall. These observations strongly indicate that the majority of the waves are created by the action of the grid directly, as opposed to, say, hypothetical energy transfer from turbulent eddies. This is supported by the dispersive spectral energy being similar between case A1 and A2 (see Fig. 11, middle row), despite the latter being more turbulent.

There is a marked asymmetry between free waves propagating upstream vs downstream in the mean-flow-following reference frame (i.e. slower vs faster than *U* in the laboratory frame, respectively). As qualitatively evident in the linear-scale plots of the surface spectra in the middle row of Fig. 11, the PSD is higher along the lower dispersion branch for small $\kappa _x$ (longer waves) than along the higher branch. For a clearer, yet only semi-quantitative illustration we compare the amount of spectral signal close to the two dispersion branches, by integrating the spectra shown in the top two rows of Fig. 11 over a band $\pm 25$ rad/s around each dispersion curve. The result is shown in the bottom row of Fig. 11. Note that this is a very rough quantity meant only for qualitative illustration, yet the asymmetric trend is clearly evident. This is another hint that the majority of the waves are made by a source which is in motion relative to the free surface, for instance, the active grid/surface plate at the inlet (at rest in the laboratory frame). That the asymmetry between the two branches is more pronounced in case ST might be due to omnidirectional scattering of waves on turbulent eddies (Smeltzer et al. 2023) that the increase in turbulence is accompanied with additional local and isotropic wave generation through a Philips-type mechanism (see, e.g., Teixeira and Belcher (2006)). A more detailed investigation of the source of wave energy is an interesting prospect for future experiments.

## Conclusions

When using an active grid to generate turbulence while keeping the mean velocity unchanged, we find that the gas transfer rate increases with turbulence intensity. Comparing our active grid cases A1 and A2 to our no-grid reference case denoted REF, we find increases of more than 30% for the gas transfer rate coefficients *k* in Table 1. Between REF and the static grid case ST, there is the increase in *k* of 15%.

From comparing the statistics of the flow and the surface in Table 1, with the values of *k* in that same table, we conclude that the intensity of vertical velocity fluctuations $w'/U$ has the largest influence on *k*. This is in line with conclusions from other authors based on experiments at lower (turbulent) Reynolds numbers in zero-mean flow facilities (Herlina and Jirka 2008; Variano and Cowen 2013). The physical explanation for the relevance of the vertical fluctuations is straightforward, as the transport of dissolved oxygen from near-surface layers to deeper layers in the liquid is in essence what defines the gas transfer problem. In this context, we might have expected to find a relation between $L_{ww}$ and *k*, which we do not convincingly find in our data. This motivates future studies that should resolve the flow field near the free surface with respect to the local depth below the surface. Seeing the wavy nature of the flow and the fluctuating water level height as a result of the varying blockage of the active grid, this can only be achieved by accurately tracing the free surface.

To capture the possible influence of surface deformations on the gas transfer, the surface area and spatio-temporal spectra of the surface deformations are obtained from synthetic schlieren measurements. The increase in surface area is less than 0.25% and, seeing that the rate of transport across an interface is proportional to the area of that interface, it can be ruled out as a significant contributor to the enhanced gas transfer rates of over 30%. Waves and imprints of convected turbulent structures appear in different ways in the spatio-temporal spectrum of the surface elevation, allowing us to conclude that the majority of surface deformations are due to dispersive surface waves. An asymmetry between downstream and upstream propagating waves indicates that these are mostly generated upstream, although there is also evidence of wave directional diffusion from scattering or, possibly, waves emerging locally from turbulence near the surface. Thus, our experiment illustrates, within the parameter space investigated, that the turbulence in the water plays a stronger role than the surface itself on gas transport.
